# Racial/Ethnic disparities in drug use during the COVID 19 pandemic: Moderating effects of non-profit substance use disorder service expenditures

**DOI:** 10.1371/journal.pone.0270238

**Published:** 2022-06-30

**Authors:** Hyunjung Ji, Su Hyun Shin, Annah Rogers, Jessica Neese, Hee Yun Lee

**Affiliations:** 1 Department of Political Science, University of Alabama, Tuscaloosa, Alabama, United States of America; 2 Department of Family & Consumer Studies, University of Utah, Salt Lake City, Utah, United States of America; 3 School of Social Work, University of Alabama, Tuscaloosa, Alabama, United States of America; University of Jyvaskyla, FINLAND

## Abstract

The COVID-19 pandemic influenced individuals’ anxiety and depression across the United States over a short period, and some Americans relied on drugs for coping. This study examines American adults’ drug use trajectories in response to changing anxiety and depression levels during the COVID-19 pandemic and the moderating role of substance use disorder (SUD) services provided by non-profit facilities in anxiety/depression-induced drug use. Heterogeneity in such relationships is further explored based on race/ethnicity. This study used a nationally representative sample of 1,176 American adults who reported drug use between May 1, 2020, and June 30, 2021. Using individual-fixed effects Poisson estimators, the current study empirically modeled drug use changes according to changing anxiety/depression levels. Interaction terms between anxiety/depression levels and per capita spending by non-profit SUD facilities were used to explore the moderating effect of SUD service expenditures. Racial/ethnic disparities were explored in subgroup analyses on non-Hispanic White, non-Hispanic Black, Hispanic, and non-Hispanic Asian samples. We found more frequent drug use in response to elevated anxiety and depression during the COVID-19 pandemic. Greater spending on SUD service by non-profit facilities at the county level was associated with reduced drug consumption associated with anxiety and depression, with greater benefits for racial/ethnic minorities. Findings provide important policy implications for distributing public funds for non-profit SUD facilities for mitigating SUD risks, especially among racial/ethnic minorities.

## Introduction

Individuals often rely on substances to adapt to and cope with stress [1]. While substance use can help people suppress stress and consequent mental health issues in the short term, it is not a resilient behavior because it can develop into substance use disorders (SUDs) [2]. Indeed, SUDs have become a public health crisis in the United States and globally, with the opioid epidemic driving-related deaths and hospitalizations [3, 4]. However, SUD treatment rates remain low across the United States, partially due to the lack of extant SUD care capacities and insufficient infrastructure to provide care for those in need [5, 6]. Racial/ethnic minorities’ access to the SUD prevention and treatment services (hereafter referred to as SUD services) is further reduced by the systematic inequality in the geographical distribution of the SUD services, with fewer SUD facilities located in communities with higher percentages of Black and Hispanic residents [5, 7, 8].

Availability of SUD services in communities can improve access to the service and timely and continuous delivery of treatment and care, which are essential to address emerging and ongoing SUD problems [7, 9]. Despite the important potential of SUD services at the community level to mitigate the risk of SUDs, we have little understanding of this relationship. A longitudinal analysis is needed to examine how individuals change their substance use in response to emerging or elevated mental health issues and to what extent such substance use is moderated by SUD services spending levels in their communities. The COVID-19 pandemic provides a unique opportunity to examine such a relationship.

First identified in 2020, the novel coronavirus (COVID-19) spread quickly worldwide and was ultimately labeled as a pandemic leading to significant social, economic, and personal costs. The pandemic has contributed to elevated stress levels and worsened depression and anxiety symptoms worldwide. The United States was hit especially hard by the pandemic with the highest number of COVID-19 related deaths in the world [9–12]. As Americans have faced heightened levels of stress and depression due to the pandemic, many have increased substance use as a coping mechanism [10, 11, 13].

In this study, we aim to examine the extent to which individuals have changed their drug use in response to changing anxiety and depression levels during the COVID-19 era and to what extent such anxiety/depression-related drug use is moderated by non-profit facilities’ SUD service provisions in their residential counties. We also explore heterogeneity in the moderating effect of nonprofit SUD facilities’ expenditures according to race/ethnicity.

To our best knowledge, this is one of the first empirical studies examining to what extent individuals with the risk of SUD problems benefit from SUD services in their community while also uncovering racial/ethnic disparities in experiencing such benefits. While some previous studies provided important evidence for racial/ethnic disparities in the geographical distribution of the SUD facilities, they focused on racial/ethnic characteristics at the community level [5, 7, 14]. Other studies examined the factors associated with receiving SUD treatment at the individual level [15, 16]. However, little is known about how substance use behaviors change with SUD services spending levels in the community. This study provides important implications for public health policy with empirical evidence of the benefit of SUD services provided by non-profit facilities for mitigating residents’ SUD risks, especially for racial/ethnic minorities.

## Methods

### Data

To explore the moderating effect of SUD service expenditures in communities on the relationship between individuals’ anxiety/depression and drug use, the present study used an individual-level panel dataset of the Understanding America Study (UAS) COVID-19 survey. On March 10, 2020, the University of Southern California (USC) began administering a bi-weekly online survey to approximately 7,500 American adults aged 18 or older that asked about their perceptions, behaviors, and health conditions related to COVID-19 [17]. To ensure the representativeness of the sample, the UAS used an address-based, multi-stage probability sampling method [18]. First, the survey retrieved approximately 135 million residential addresses from U.S. Postal service’s Computerized Delivery Sequence (CDS) file, which covers almost 100% of U.S. households [18]. The UAS then randomly drew its sample using a sequential probability sample batching [18]. Specifically, after the first round of random selection, subsequent batches were selected by assigning unequal sampling probabilities to each zip code to make the sample more representative across key demographic characteristics such as sex, age, education, etc. [18]. For instance, if the sample drawn in the first batch underrepresented women aged 65 or older, the UAS assigned a higher probability on zip codes with a higher proportion of women aged 65 or older in subsequent batches. This sampling process is implemented for multiple iterations until it achieves the sample representativeness [18].

To mitigate sampling attribution associated with an online survey, the UAS provided tablets and internet subscriptions to those without them [17]. Researchers show that the sample quality is as good as ones collected from traditional surveys conducted face-to-face and/or via phone, such as the Health and Retirement Study [19]. Based on the technical reports provided by the UAS, the participation rates tended to decline over time ranging from 76–94% with the average rate of 83% (see https://uasdata.usc.edu/index.php). The current study used the 4–29 waves of the UAS between May 1, 2020, and June 30, 2021. The first three waves were excluded in this study because they do not include some of the key covariates (e.g., resilience).

Individual-level data were merged to various county-level data (e.g., SUD facilities) based on the UAS respondents’ county of residence. Because geographic information below the state-level is restricted data, the data administrator linked external data to the UAS based on a respondent’s county of residence and then shared the merged data after eliminating any identifiable geographical information of respondents. To prevent researchers from identifying each respondent’s county of residence, the data administrator further masked some variable values (e.g., extreme values) by aggregating them into fewer categories or rounding them off.

### Variables

#### Anxiety and depression

To measure anxiety and depression, the UAS used the four-item Patient Health Questionnaire (PHQ-4). The PHQ-4 asks how many days a respondent has been bothered by the following problems over the past two weeks: feeling nervous, anxious, or on edge; not being able to stop or control worrying; feeling down, depressed, or hopeless; and little interest or pleasure in doing things. The responses were ’not at all (zero),’ ’several days (one),’ ’more than half the days (two), and ’nearly every day (three).’ The Cronbach alpha for the four items was 0.92, ensuring the internal reliability of the measure. A total PHQ-4 score was calculated by summing up the four-item (score 0–12). To account for potential nonlinearity of the relationship between anxiety/depression and outcomes, the total PHQ-4 score was categorized into four levels: normal (score 0–2); mild (score 3–5); moderate (score 6–8); and severe (score 9–12) [20].

#### SUD service expenditures

As a proxy for SUD service provision at the county level, the current study used the total expenditure of non-profit SUD facilities in each county, which was then standardized by county population [21]. This study primarily focused on non-profit SUD facilities to measure SUD service expenditures for two reasons. First, with our primary objective to inform policymakers about how to approach SUD risks via public funding and policy, we can draw more relevant policy implications from the effect of non-profit facilities than from those of for-profit facilities. Non-profit SUD facilities have heavily relied on public funds for their service provision [14], which is not the case for for-profit facilities. In 2020, 73.2% of non-profit SUD facilities received public funds for SUD treatment programs, whereas only 17.7% of private for-profit facilities did [22]. With public funds, non-profit SUD facilities have also played a significant role in serving their clients with limited accessibility, such as low-income, racial/ethnic minorities [14, 23]. Findings from the publicly funded non-profit facilities can provide important implications for the distribution of public funds to underserved populations, especially racial/ethnic minorities. Second, the financial data was available only for non-profit SUD facilities operations [24].

The expenditure information of non-profit SUD facilities was retrieved from the 2017 National Center for Charitable Statistics (NCCS) core files, including the information listed on the Form 990 that every non-profit organization must file to the U.S. Internal Revenue Service (IRS) each fiscal year for tax exemption. The NCCS data include various organizational information, such as financial information, National Taxonomy of Exempt Entities (NTEE) codes corresponding to primary activities, and geographical information of each non-profit organization. SUD facilities were identified if a non-profit organization’s NTEE code was F20 (Substance Abuse Dependency, Prevention & Treatment), F21 (Substance Abuse Prevention), or F22 (Substance Abuse Treatment). We aggregated expenditures spent by all the SUD facilities located in each county and divided them by the county population with selected non-profit SUD facilities. The latest available NCCS data was from 2017 because many non-profits submit their required reporting form to the IRS with one- or two-year delays, and their more recent information has not yet been updated in the data repository.

To mask the exact value of expenditures per capita that otherwise can be used to identify the geographical location of respondents, the value was rounded to the nearest one if it was less than 10; to the nearest 10 if it was greater than or equal to 10 but less than 100; to the nearest 100 if it was greater than or equal to 100 but less than 300. If the value of expenditure per capita was greater than or equal to 300, it was consolidated at 300.

#### Drug use

The outcome variable of the current study was drug use. Drug use behavior was assessed using the question asking, "out of the past seven days, what is your best estimate of the number of days that you used recreational drugs other than alcohol or cannabis product?" The responses ranged from zero to seven.

#### Race/Ethnicity

To explore heterogeneity in the moderating effect of SUD service availability by race/ethnicity, the present study conducted subgroup analyses by splitting the sample by non-Hispanic White, non-Hispanic Black, Hispanic, and non-Hispanic Asian adults. In the subgroup analyses, other racial categories, such as American Indian or Alaska Native, Hawaiian/Pacific Islander, and mixed races, were excluded from our analyses. We hereafter denote ‘non-Hispanic’ White, Black, and Asian adults by White, Black, and Asian adults.

#### Covariates

The present study controlled for various time-variant socio-demographic factors, such as marital and employment status, household income, health insurance ownership, number of household members, and the presence of children under 18 in the household. It is noteworthy that age and educational attainment were treated as time-invariant because they were absorbed in individual-fixed effects due to little variations during our short study period.

We further controlled for psychological factors potentially associated with anxiety/depression and substance use, including resilience and perceived stress. The Brief Resilience Scale (BRS) was used to assess resilience [25]. The six items were "I tend to bounce back quickly after hard times," “I have a hard time making it through stressful events,” “It does not take me long to recover from a stressful event,” “It is hard for me to snap back when something bad happens,” “I usually come through difficult times with little trouble,” and “I tend to take a long time to get over setbacks in my life.” The responses to each item ranged from one (strongly disagree) to five (strongly agree). A composite score was created by summing up the six items after reverse-coding the three negatively worded items. The higher the score is, the more resilient a person is. The Cronbach alpha for the six items was 0.87. Perceived stress was assessed using the Perceived Stress Scale 4 (PSS-4) [26]. The UAS asks, in the past two weeks, how often a respondent has felt: that a respondent was unable to control the important things in his/her life; confident about his/her ability to handle personal problems; that things were going his/her way; and difficulties were piling up so high that he/she could not overcome them. The responses to each item ranged from one (never) to five (very often). After reverse-coding the second and the third items, a total score was calculated by summing up the four items. The Cronbach alpha of the four items was 0.71.

Considering the unique context of COVID-19, we also controlled for the community- and individual-level COVID-19 related risk factors that might trigger anxiety and depression. As community-level risk factors, we used daily county-level new case and death rates per capita, both of which were retrieved from the Johns Hopkins University Center for Systems Science and Engineering (JHU CSSE) data depository [27]. Such data were merged to the UAS data based on respondents’ county of residence and survey dates. As noted earlier, to prevent researchers from reverse-engineering geographic information about respondents, the value of both variables was masked by rounding the new case rate to the nearest 0.005 and the death rate to the nearest 0.0005. To assess individual perceived risk of COVID-19, three items were used: the percent chance a respondent will get the coronavirus in the next three months; will die from the coronavirus; and will run out of money because of the coronavirus in the next three months. The value of the three variables ranged from zero to one.

We further controlled for COVID-19 related government benefits and state-level health guidelines that potentially mitigate or exacerbate anxiety and depression. The two indicators of receiving governmental benefits were whether a respondent or anyone in his/her household received economic stimulus funds in the past month and whether he/she received unemployment insurance benefits in the past two weeks. Three statewide health mandate indicators were also controlled: whether a respondent lived in a state with stay-at-home; non-essential business closure; or overnight business curfew orders on survey dates. The information about statewide orders was retrieved from OpenICPSR data depository [28].

### Empirical models

To model whether spending on SUD services played a moderating role in the relationship between anxiety/depression and drug use, the following individual-fixed effects Poisson model was estimated:

$$\;log\;log\;\left(Drug_{it}\right)=\beta_{0}+\beta_{1}Dep_{it}+\beta_{2}Dep_{it}\times SUD_{c}+\beta_{3}X_{it}+i_{i}+t_{t}+\varepsilon_{it}$$ (1)

This method is appropriate because the dependent variable of *Drug*_*it*_ is the number of days using recreational drugs by individual *i* in *t*. Poisson models keep only those individuals whose responses to the outcome variable (i.e., drug use) vary over time in the sample. Therefore, those who did not consume any drugs during the study period were excluded from the sample. The individual-fixed effects model enabled us to model changes in drug use in response to changes in anxiety/depression levels over time by controlling for time-invariant individual characteristics given SUD service expenditures in the community. For anxiety/depression levels (*Dep*_*it*_), we included three indicators denoting ‘mild,’ ‘moderate,’ and ‘severe’ conditions by setting a ‘normal’ level as the reference group. *SUD*_*c*_ indicates pre-existing level of per capita expenditures by non-profit SUD facilities in county c prior to the pandemic. Eq (1) does not include the standalone term of *SUD*_*c*_ and its coefficient because the effect of the variable is likely to be absorbed in individual-fixed effects of *i*_*i*_, with a county of residence not changing among a majority of the UAS sample. *X*_*it*_ denotes a vector of time-variant individual- and household characteristics. *i*_*i*_ and *t*_*t*_ are individual- and time fixed effects. *ε*_*it*_ denotes an idiosyncratic error term. The coefficient of interest is *β*_2_. It should be noted that time-invariant individual-characteristics, such as race/ethnicity, were absorbed into individual-fixed effects (*i*_*i*_). We further estimated Eq (1) by four racial/ethnic groups: White, Black, Hispanic, and Asian to identify differences in the relationships described above. In all statistical analyses, we sample-weighted the estimates to present the results in a more nationally representative way.

## Results

### Sample characteristics

Table 1 presents characteristics of the pooled sample and by race/ethnicity. The descriptive statistics were obtained using observations (wave-respondents) rather than unique respondents to present weighted estimates for panel data. Therefore, the descriptive statistics of time-variant factors reflect differences across groups and changes within the same individual over time. The summary statistics for time-invariant factors are the prevalence rate of each category. It should be noted that differences in the means and the proportions of each characteristic among racial/ethnic groups were not statistically tested. Therefore, the study simply compares these estimates across subgroups without making any statistical inferences.

**Table 1:** Sample characteristics.

	Pooled	White	Black	Hispanic	Asian
Dependent variables					
Days of drug use	0.54	0.55	0.54	0.55	0.40
	(1.57)	(1.62)	(1.52)	(1.55)	(1.24)
Days of drug use if any	3.71	3.94	3.66	3.38	3.05
	(2.28)	(2.33)	(2.09)	(2.28)	(1.90)
Explanatory variables					
SUD facility expenditure per capita ($)	20.63	20.45	14.38	24.17	27.68
	(35.30)	(38.74)	(22.79)	(29.28)	(48.03)
PHQ-4					
Normal	64.92	63.13	70.50	64.63	65.15
Mild	19.09	19.00	16.76	22.00	19.61
Moderate	8.31	8.73	7.24	8.71	4.50
Severe	7.69	9.14	5.50	4.67	10.74
Covariates					
Age	47.31	50.01	47.75	40.06	44.95
	(16.54)	(17.15)	(15.19)	(12.66)	(19.71)
Education					
Less than HS	11.59	12.41	16.33	7.20	6.22
HS	35.41	38.37	33.95	33.30	20.12
SC	27.56	23.87	31.65	35.35	27.11
BA	16.94	18.18	8.25	15.45	34.36
Graduate	8.49	7.17	9.82	8.70	12.18
Marital status					
Married	46.32	52.50	30.84	45.13	45.08
Sep./div./wid.	22.46	23.75	19.10	20.81	21.45
Never married	31.23	23.75	50.05	34.06	33.46
Employment status					
Unemployed	17.88	12.21	22.83	26.18	21.18
Employed	56.38	51.89	58.16	66.87	57.00
Retired	25.74	35.90	19.00	6.96	21.83
Household income					
$0-$29,999	39.47	36.76	58.16	35.29	19.39
$30K-$59,999	25.21	25.85	22.69	25.65	29.59
$60K-$99,999	19.15	20.60	8.70	22.17	21.33
$100K+	16.17	16.79	10.45	16.89	29.69
No. of household members	1.67	1.53	1.60	2.21	1.55
	(1.46)	(1.41)	(1.51)	(1.50)	(1.14)
Have child <18	26.78	21.06	29.94	43.93	16.44
Health insurance ownership	81.53	84.21	78.34	74.97	90.79
Brief Resilience Scale (BRS)	20.33	20.49	20.44	20.16	18.18
	(4.41)	(4.87)	(3.76)	(3.47)	(3.83)
Perceived Stress Scale 4 (PSS-4)	9.74	9.51	10.01	9.99	10.28
	(3.37)	(3.56)	(3.10)	(3.00)	(3.05)
COVID-19 new case rate per capita	0.04	0.04	0.05	0.05	0.04
	(0.04)	(0.04)	(0.04)	(0.04)	(0.04)
COVID-19 death rate per capita	0.0009	0.0008	0.0011	0.0011	0.0007
	(0.0009)	(0.0008)	(0.0009)	(0.0011)	(0.0007)
Probability of death due to COVID-19	0.22	0.22	0.27	0.20	0.18
	(0.27)	(0.28)	(0.27)	(0.24)	(0.25)
Probability of COVID-19 infection	0.24	0.23	0.26	0.27	0.23
	(0.23)	(0.23)	(0.25)	(0.24)	(0.24)
Probability of running out of money due to COVID-19	0.21	0.17	0.28	0.26	0.18
	(0.28)	(0.26)	(0.30)	(0.28)	(0.27)
Stimulus check	16.76	16.51	16.87	19.28	9.92
Unemployment insurance	7.43	5.64	5.16	13.85	8.04
Stay-at-home order	12.55	9.15	5.70	23.09	31.71
Non-essential business closure	2.56	2.81	2.13	2.18	3.46
Business curfew	12.03	13.22	10.06	10.80	9.35
Obs.	22,302	12,544	2,446	4,528	1,401
N	1,176	634	133	267	69

*Note*. 4–29 waves of UAS. Sample weighted estimators. For continuous variables, we present means and standard deviations in parentheses. For categorical variables, we present proportions of people who are included in each category.

Related to our key dependent and independent variables, the average number of days using drugs was similar across racial/ethnic groups and the pooled sample (avg. = 0.54–0.55), except for Asians (avg. = 0.40), when including the days without using drugs. Conditional on any drug use, the average days of drug consumption was highest among Whites (avg. = 3.94) and lowest among Asians (avg. = 3.05). The community’s average non-profit SUD facilities expenditure per capita was highest among Asians (avg. = 24.17) and lowest among Blacks (avg. = 14.38). The percentage of observations that scored moderate or severe anxiety/depression was highest among Whites (17.87%) and lowest among Blacks (12.74%).

In reporting some important covariates, the unemployment rate during the study period was highest among Hispanics (26.18%), followed by Blacks (22.83%) and Asians (21.18%). The percentage of observations earning household incomes of at least $60,000 was highest among Asians (51.02%), followed by Hispanics (39.06%) and Whites (37.39%). Health insurance ownership was also highest among Asians (90.79%), followed by Whites (84.21%).

The average BRS score was similar across racial/ethnic groups and the pooled sample, except for Asians. Asian observations scored the lowest in the BRS (avg. = 18.18). Further, Asian observations scored the highest on the PSS-4 scale, with an average score of 10.28. Conversely, the average PSS-4 score was lowest among White observations (avg. = 9.51).

COVID-19 new case rates per capita were similar across racial/ethnic groups and the pooled sample, while death rates per capita were higher among Blacks and Hispanics. Consistent with the objective data on higher new case and death rates in these communities, the perceived risk of infection and death due to COVID-19 was highest among Blacks. As the data on higher unemployment rates, Black and Hispanic observations reported a higher perceived risk of running out of money due to COVID-19 than other groups. Due to their economic insecurity, a greater proportion of Hispanic observations received stimulus checks (19.28%) and unemployment insurance benefits (13.85%).

### County-level spending by nonprofit SUD facilities

**Fig 1:**
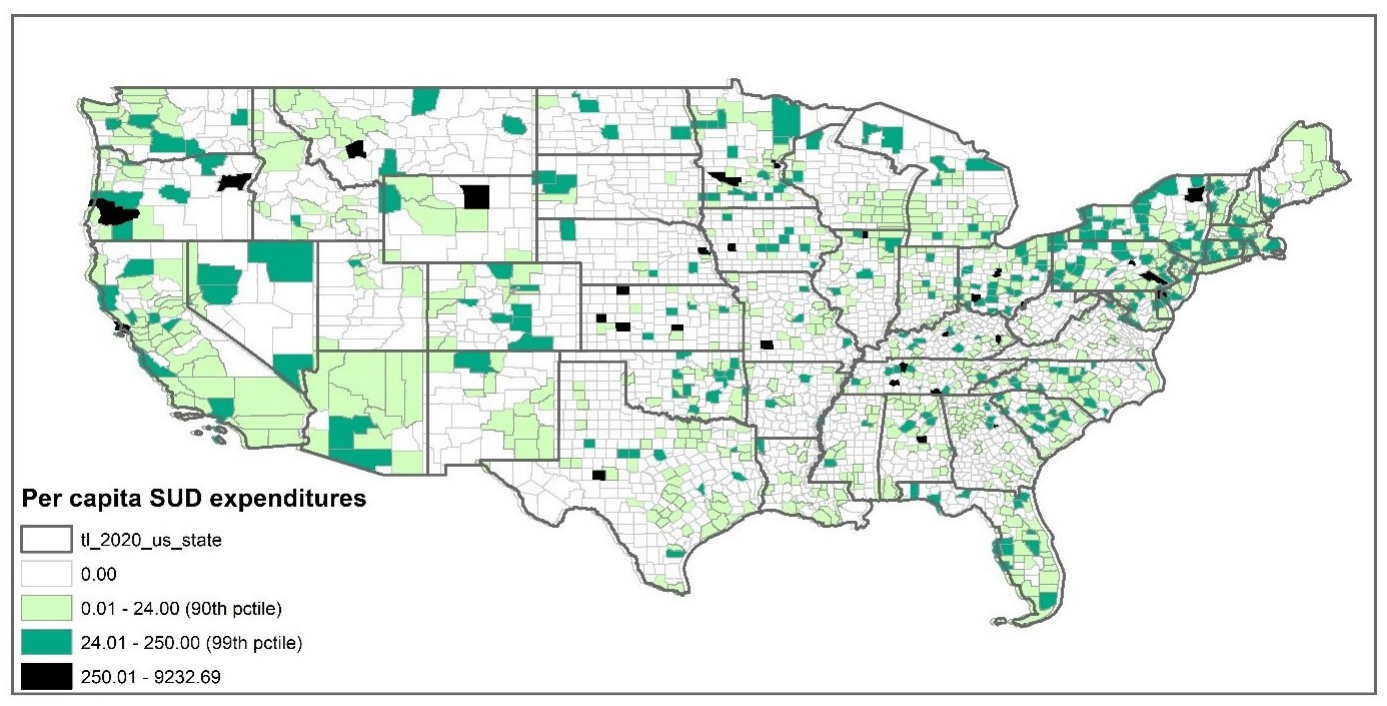
County-level distribution of nonprofit SUD facilities’ expenditure per capita.

Fig 1 presents the geographical distribution of the per capita spending by nonprofit SUD facilities across U.S. counties. As shown in Fig 1, in 2017, many counties with especially high levels of SUD expenditures (i.e., more than $ 250 per capita) were located in Northeastern states, such as New Jersey and Pennsylvania. Oregon and Tennessee also had some counties with high levels of SUD spending per capita. By contrast, many states with especially high proportions of counties with no expenditures of non-profit SUD facilities were concentrated in the South, such as West Virginia (83% of counties had zero spending), Arkansas (84%), and Mississippi (79%). In the Midwest, there was an interesting dichotomy, with counties in some states having the lowest SUD expenditures while those in others had the highest. For example, counties in Minnesota, and Ohio had some of the highest SUD expenditures, while many counties in North Dakota (92%), Nebraska (91%), South Dakota (90%), Illinois (81%), Missouri (80%) had zero spending. Cummings et al. provided a few empirical investigations for the geographical variations in SUD facility availability across U.S. counties [4]. Using a binary indicator for the presence of at least one outpatient SUD treatment facility in a county, they found counties with lower presences of SUD facilities were concentrated in Southern and Midwestern states. Our findings about the levels of SUD expenditures across the United States are somewhat consistent with the geographical distribution of the SUD facility availability measure used by Cummings et al. Even though they used a slightly different measure for SUD service from ours (i.e., per capita expenditure by non-profit SUD facilities), their findings are consistent with the geographical distribution of our SUD service expenditure measure.

### Main results

Table 2 presents the sample weighted estimates from individual-fixed effects Poisson models using the pooled sample (Column I) and race/ethnicity (Columns II-IV). Compared to the times with a normal level, among the pooled sample, increased anxiety/depression to mild, moderate, and severe levels related to 41.03, 65.44, and 90.45 percent more days of drug use, respectively. In aggregate, it seemed that greater levels of spending on SUD services in the community did not relate to reduced drug consumption for those who experienced elevated anxiety/depression. With higher spending by non-profit SUD facilities per capita, those who experienced increased anxiety/depression to moderate levels consumed drugs 0.25 percent more days than those with a normal anxiety/depression level.

**Table 2:** Effects of SUD facility spending and anxiety/depression on drug use.

	(I) Pooled		(II) White		(III) Black		(III) Hispanic		(IV) Asian	
	IRR (Std. Err.)	[95% CI]	IRR (Std. Err.)	[95% CI]	IRR (Std. Err.)	[95% CI]	IRR (Std. Err.)	[95% CI]	IRR (Std. Err.)	[95% CI]
PHQ-4										
Mild	1.4103*** (0.0497)	[1.32, 1.51]	1.3739*** (0.0680)	[1.25, 1.51]	1.5418*** (0.1358)	[1.30, 1.83]	1.4937*** (0.1489)	[1.23, 1.82]	1.2089 (0.2456)	[0.81, 1.80]
Moderate	1.6544*** (0.0739)	[1.52, 1.81]	1.5613*** (0.0960)	[1.38, 1.76]	1.9065*** (0.2946)	[1.41, 2.58]	2.5552*** (0.2797)	[2.06, 3.17]	1.6288 (0.6230)	[0.77, 3.45]
Severe	1.9045*** (0.0997)	[1.72, 2.11]	1.7721*** (0.1206)	[1.55, 2.02]	2.5193*** (0.4023)	[1.84, 3.45]	2.4918*** (0.4018)	[1.82, 3.42]	4.6546*** (1.9213)	[2.07, 10.45]
PHQ-4 × SUD facility										
Mild ×	0.9989 (0.0007)	[1.00, 1.00]	0.9990 (0.0009)	[1.00, 1.00]	0.9944 (0.0031)	[0.99, 1.00]	0.9930* (0.0033)	[0.99, 1.00]	1.0092* (0.0041)	[1.00, 1.02]
Exp. Per										
cap										
Moderate	1.0026* (0.0010)	[1.00, 1.00]	1.0058*** (0.0013)	[1.00, 1.01]	0.9770** (0.0071)	[0.96, 0.99]	0.9872*** (0.0036)	[0.98, 0.99]	0.9927 (0.0175)	[0.96, 1.03]
× Exp.										
Per cap										
Severe ×	0.9978 (0.0015)	[0.99, 1.00]	1.0026 (0.0022)	[1.00, 1.01]	0.9887** (0.0039)	[0.98, 1.00]	0.9871** (0.0042)	[0.98, 1.00]	0.9272** (0.0228)	[0.88, 0.97]
Exp. Per										
cap										
Obs.	22,302		12,544		2,446		4,528		1,401	
N	1,176		634		133		267		69	

*Note*. Sample weighted estimators. Individual-fixed effects Poisson regression estimators. IRR = Incidence Rate Ratio; CI = Confidence Interval; PHQ-4 = Patient Health Questionnaire-4; SUD = Substance Use Disorder; Exp. Per cap = expenditure per capita. All the covariates listed in the method section are controlled for.

* *p*<0.05;

** *p*<0.01;

*** *p*<0.001.

The current study found heterogeneity in the moderating effects of non-profit SUD facilities’ expenditures on the relationship between anxiety/depression and drug use across different racial/ethnic groups. White adults showed similar patterns to the pooled sample (Column II). Relative to the normal level times, White adults with anxiety/depression elevated to mild, moderate, and severe levels used drugs 37.39, 56.13, and 77.21 percent more days. For an additional $1 per capita spent by non-profit SUD facilities in their county of residence, White adults whose anxiety/depression increased to a moderate level used drugs 0.58 percent more days.

Both Black and Hispanic adults showed more frequent recreational drug use when they experienced elevated anxiety/depression to mild (54.18% & 49.37%), moderate (90.65% & 155.52%), and severe (151.93% & 149.18%) levels (Columns III & IV). However, with higher SUD service expenditure per capita in the community, both groups exhibited lower drug consumption even with elevated anxiety/depression. Specifically, Black adults’ drug consumption was 2.30 and 1.13 percent lower for those whose anxiety/depression levels increased to moderate and severe levels if their county had higher levels of spending on SUD services. Similarly, with higher SUD service expenditure per capita in the community, Hispanic adults’ drug consumption was lowered by 0.70, 1.28, and 1.29 percent among those who experienced elevated anxiety/depression to mild, moderate, and severe levels.

Asian adults’ drug consumption did not change even when they experienced elevated anxiety/depression to mild and moderate levels. However, those whose anxiety/depression elevated to a severe level showed a 365.46 percent increased drug use compared to when they showed a normal level (Column IV). With greater levels of spending on SUD services by non-profit facilities in the community, Asian adults with elevated anxiety/depression to mild reported 0.92 percent higher drug use relative to the times with a normal level, while those with increased anxiety/depression to a severe level related to a 7.28 percent lower drug consumption with higher SUD service spending.

**Fig 2:**
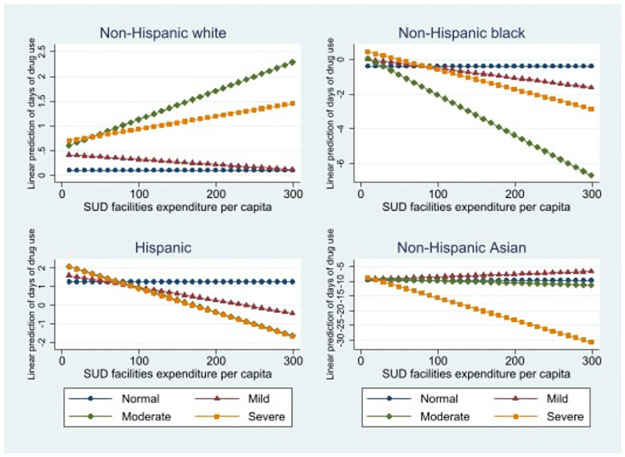
Linear prediction of drug use by racial/ethnic groups.

Fig 2 presents the predicted margin plots of anxiety/depression levels on drug use with higher per capita expenditure of non-profit SUD facilities by racial/ethnic groups. Consistent with the findings presented in Table 2, the figure better illustrates the moderating role of SUD service expenditures in drug use related to elevated anxiety/depression.

As a robustness check, we ran additional analyses by excluding the health insurance ownership variable from our original models (Eq 1). Financial characteristics, such as income and insurance status, are key factors affecting an individual’s access to SUD services [21]. Under the unprecedented levels of economic shocks during COVID-19 especially, many Americans lost their employment-based health insurance coverages [29], which may have influenced their access to SUD service. Since the original models already controlled for individual income and other time-invariant individual characteristics, the additional control for health insurance ownership may constrain our ability to capture whether nonprofit SUD facilities disproportionately benefit people without health insurance. Therefore, we re-ran our original models after excluding the health insurance ownership variable to see how the results change across different racial/ethnic groups.

The results from the models without health insurance ownership adjustment (available in S1 Table) showed almost the same results as our original models but revealed more significant effects for Black adults than other groups. More specifically, the interaction term between the mild level of anxiety/depression and the non-profits’ SUD spending became significant for Blacks in the negative direction, which was not significant when accounting for the health insurance ownership in the original model. The additional result indicates that Black adults may have disproportionately benefited from non-profit SUD facilities when they experienced a mild level of anxiety/depression and did not have health insurance.

## Discussion

Even though responding to the SUD problem has become an urgent public health agenda item in the U.S., extant SUD facilities cannot meet the service need of SUD patients [5, 6, 30]. For-profit SUD facilities have grown rapidly over the last years, but these facilities are less likely to be accessible among socioeconomically disadvantaged people [30] due to the higher concentration of SUD facilities in high-income, White communities [14]. The expansion of Medicaid and public funds for SUD service under the Affordable Care Act is expected to make SUD service more accessible, especially for those who are currently underserved, thereby mitigating SUD risks [3, 5, 30]. However, people can only benefit from the expanded SUD service if related facilities and resources are available in their communities [2].

Focusing on the non-profit SUD facilities whose operations are primarily funded by public funds, this study empirically examined the extent to which county-level SUD service expenditures benefitted individuals who suffered from elevated anxiety/depression during the pandemic and otherwise relied on drug use for unhealthy coping. We further explored if the benefits of SUD service expenditures on drug use related to anxiety/depression varied across different races/ethnicities. While we chose non-profit SUD facilities for theoretical and empirical reasons, our results should be understood with caution as our results are limited to non-profit SUD facilities only and can be different from results from for-profit SUD facilities or entire SUD facilities.

Overall, our findings suggest that American adults, on average, were likely to use drugs more frequently when experiencing increased anxiety/depression. Although it was not statistically tested, the effect size seemed to be larger among Blacks, Hispanics, and Asians than Whites. The more severe impacts of the pandemic for Blacks and Hispanics than Whites, such as higher death rates, more limited access to testing, and greater economic loss, might have contributed to this greater drug use frequency among these racial/ethnic groups [10, 31]. Asians were also adversely impacted by COVID-19 and Anti-Asian Racism [32]. During the pandemic, Asians were stereotyped as the reason for the COVID-19 pandemic, and the virus was labeled “China virus” [32]. Exposure to such racism and discrimination and the consequent hate crimes magnified stress, anxiety, and depression among Asians [32].

We found that spending on SUD services by non-profit facilities in the community was related to reduced drug use due to anxiety/depression amongst racial/ethnic minority groups. Our subgroup analysis indicates that Whites with elevated anxiety/depression who lived in counties with higher SUD expenditures by non-profits reported more frequent drug use. However, in general, all other racial/ethnic groups showed less frequent drug use when they lived in counties with higher SUD expenditures by non-profits, even when their anxiety/depression rose to moderate or severe levels during the pandemic. These results indicate that the risk of drug misuse for coping with anxiety/depression was mitigated by community-level SUD services provided by non-profit facilities for racial/ethnic minorities but not Whites during the pandemic.

More specifically, drug use by Black adults who experienced elevated anxiety/depression to moderate or severe levels was moderated by higher levels of spending on service provision by non-profit SUD facilities in their county of residence than other counties. The moderating effects of non-profit SUD service were similar but more pervasive for Hispanics than Blacks. Hispanics’ drug use associated with elevated anxiety/depression to the mild level also benefited from SUD service expenditures, whereas Blacks did not.

Accumulated evidence has shown more limited access to SUD services among Blacks and Hispanics than Whites despite the similar prevalence of SUD between the groups [32]. Our findings on the moderating effect of non-profit’s SUD service expenditures provide optimistic views because, contingent on the presence of non-profit SUD facilities in their community, greater levels of spending on service provision by the facilities was associated with reductions in these racial/ethnic groups’ drug use related to mental issues. This finding was robust even after adjusting for financial characteristics, such as insurance ownership, which often affect access to SUD services [21]. As shown in the additional analyses excluding the health insurance ownership variable from our original models (available in S1 Table), nonprofit SUD facilities’ spending has more significant effects among Blacks than other racial/ethnic groups. Given non-profits’ disproportionately higher acceptance rates for low-income racial/ethnic minorities than Whites [23], Blacks and Hispanics in our current sample may be more likely than Whites to rely on the non-profit SUD facilities for economic reasons.

Unlike other racial/ethnic minorities, the results for Asians showed different patterns in terms of the moderating effects of the community-level non-profit SUD services. Greater expenditures on service provision by non-profit SUD facilities in their communities was associated with reduced drug use among Asians with elevated anxiety/depression to severe levels. However, Asians who experienced anxiety/depression increasing to mild levels showed more frequent drug use even when located in communities with greater levels of non-profit SUD service spending. Garrison et al.’s study may partially explain these findings on Asians [33]. Asian participants of the Garrison et al.’s study were likely to seek treatment only if their self-reported substance use became "severe” [33]. Asians did not seek the SUD service until their mental health and substance use issues reached a severe level. Similarly, Asians in the current sample who experienced mildly increased anxiety/depression may deem their current mental issues not severe and, thus, not seek the SUD treatment service from non-profit facilities and instead rely more on drug use. By contrast, those higher at-risk Asian respondents with a more dramatic increase in anxiety/depression may seek SUD treatment and be more likely to reduce their use of drugs.

## Conclusion

Non-profit SUD facilities have served an important role in delivering SUD treatment services [30], especially for low-income racial/ethnic minorities [23]. However, their market shares have been outpaced by private for-profit facilities. For example, between 2010 and 2020, non-profit substance use facilities saw a decrease in market share from 58% to 50% of all facilities, while private for-profits increased their market share from 30 to 41% of all facilities [22]. Our findings on the benefits of non-profits’ SUD services in moderating anxiety/depression-driven drug use among racial/ethnic minorities provide important implications for the public fund distribution for SUD and mental health policy.

True to the nature of any study that focuses on the impact of mental and behavioral health on racial/ethnic minorities, there is not a “one size fits all” approach to improve SUD or any mental health treatment admissions. Instead, practices need to be specific to each group as each group poses different barriers to seeking SUD and mental health care. Year 2 of living in a COVID-19 society is vastly approaching, and yet disparities are still evident, with some experiencing a widening gap. Although telehealth or eMedicine is highly considered to be a solution in the gap of access to SUD and other mental health care services, it has shown to have its drawbacks, especially among lower-income and rural Americans due to lack of accessibility to internet and technology devices. Special consideration of the barriers for these populations is needed before moving forward with overarching, transcultural implementation. One way of providing such special considerations is through tailored outreach that is specific to populations/communities that are more at-risk of SUD and mental health or at socioeconomic and geographical disadvantages. Furthermore, health policy initiatives that promote universal health care or more accessible substance use treatment could have the ability to improve negative outcomes of the COVID-19 pandemic.

We recognize several limitations of this study. First, the current study exclusively relied on quantitative measures for psychological and behavioral constructs, such as self-reported anxiety/depression and drug use. Given the complexity in measuring mental health and health behaviors, future research could provide more convincing evidence on the relationship among SUD service, anxiety/depression, and drug use via triangulation of quantitative and qualitative data. Qualitative data from observational or ethnographic approaches among a few representative counties and among distinct racial/ethnic groups may be a valuable addition to our empirical findings. Second, future research will also benefit by exploring the geographical disparities in spending by nonprofit SUD facilities. We found that so many counties in the south, particularly in red states in the U.S., had zero spending made by nonprofit SUD facilities, creating SUD service desert areas. The lack of access by county to a nearby SUD service facility denotes the US priorities in public health and specifically in mental and SUD care in this region. Given the racially segregated residential patterns in the U.S. by county, it would lead to even more racialized disparities. Building on the geographical disparities in the spending on SUD services, future studies should focus on exploring the factors related to the geographical variations in greater detail by using a qualitative research approach.

## Supporting information

S1 Table Additional analyses, without controlling for health insurance ownership. (DOCX)
