# Supplementary material for: Racial/Ethnic disparities in drug use during the COVID 19 pandemic: Moderating effects of non-profit substance use disorder service expenditures
Source: PLoS One. 2022 Jun 30;17(6):e0270238. doi: 10.1371/journal.pone.0270238 (PMC9246210; doi:10.1371/journal.pone.0270238)
Supplement: S1 Table — (DOCX) [file pone.0270238.s001.docx]

**Supporting information**

**S1 Table. Additional analyses, without controlling for health insurance ownership.**

|  | (I) Pooled | | (II) White | | (III) Black | | (III) Hispanic | | (IV) Asian | |
| --- | --- | --- | --- | --- | --- | --- | --- | --- | --- | --- |
|  | IRR  (Std. Err.) | [95% CI] | IRR  (Std. Err.) | [95% CI] | IRR  (Std. Err.) | [95% CI] | IRR  (Std. Err.) | [95% CI] | IRR  (Std. Err.) | [95% CI] |
| PHQ-4 |  |  |  |  |  |  |  |  |  |  |
| Mild | 1.4097***  (0.0497) | [1.32, 1.51] | 1.3721***  (0.0678) | [1.25, 1.51] | 1.5931***  (0.1397) | [1.34, 1.89] | 1.4933***  (0.1486) | [1.23, 1.82] | 1.3073  (0.2623) | [0.88, 1.94] |
| Moderate | 1.6488***  (0.0737) | [1.51, 1.80] | 1.5564***  (0.0955) | [1.38, 1.76] | 1.9557***  (0.3023) | [1.44, 2.65] | 2.5549***  (0.2795) | [2.06, 3.17] | 1.9316  (0.7268) | [0.92, 4.04] |
| Severe | 1.9000***  (0.0995) | [1.71, 2.11] | 1.7699***  (0.1204) | [1.55, 2.02] | 2.5293***  (0.4029) | [1.85, 3.46] | 2.4911***  (0.4013) | [1.82, 3.42] | 4.7191***  (1.9668) | [2.08, 10.68] |
| PHQ-4 × SUD facility | | | | | | | | | | |
| Mild ×  Exp. per  cap | 0.9989  (0.0007) | [1.00, 1.00] | 0.9990  (0.0009) | [1.00, 1.00] | 0.9934*  (0.0031) | [0.99, 1.00] | 0.9930*  (0.0033) | [0.99, 1.00] | 1.0088*  (0.0041) | [1.00, 1.02] |
| Moderate  × Exp.  per cap | 1.0027**  (0.0010) | [1.00, 1.00] | 1.0059***  (0.0013) | [1.00, 1.01] | 0.9762**  (0.0071) | [0.96, 0.99] | 0.9872***  (0.0036) | [0.98, 0.99] | 0.9899  (0.0176) | [0.96, 1.02] |
| Severe ×  Exp. per  cap | 0.9978  (0.0015) | [0.99, 1.00] | 1.0026  (0.0022) | [1.00, 1.01] | 0.9883**  (0.0039) | [0.98, 1.00] | 0.9871**  (0.0042) | [0.98, 1.00] | 0.9291**  (0.0231) | [0.88, 0.98] |
| Obs. | 22,302 | | 12,544 | | 2,446 | | 4,528 | | 1,401 | |
| N | 1,176 | | 634 | | 133 | | 267 | | 69 | |

*Note*. Sample weighted estimators. Individual-fixed effects Poisson regression estimators. IRR= Incidence Rate Ratio; CI= Confidence Interval; PHQ-4= Patient Health Questionnaire-4; SUD= Substance Use Disorder; Exp. per cap= expenditure per capita. All the covariates listed in the method section are controlled for. * *p*<0.05; ** *p*<0.01; *** *p*<0.001.
